# Supplementary material for: The Voltage-Gated Hv1 H+ Channel Is Expressed in Tumor-Infiltrating Myeloid-Derived Suppressor Cells
Source: Int J Mol Sci. 2023 Mar 25;24(7):6216. doi: 10.3390/ijms24076216 (PMC10094655; doi:10.3390/ijms24076216)
Supplement: Supplementary file 1 [file ijms-24-06216-s001.zip › ijms-2274878-supplementary.pdf]

Supplementary materials

Marco Cozzolino [1,†], Adrienn Gyöngyösi [2,†], Eva Korpos [1,3], Peter Gogolak [2], Muhammad Umair Naseem [1], Judit Kállai [2,3], Arpad Lanyi [2] and Gyorgy Panyi [1,*]

[1] Department of Biophysics and Cell Biology, Faculty of Medicine, University of Debrecen, 4032 Debrecen, Hungary
[2] Department of Immunology, Faculty of Medicine, University of Debrecen, 4032 Debrecen, Hungary
[3] ELKH-DE Cell Biology and Signaling Research Group, Faculty of Medicine, University of Debrecen, 4032 Debrecen, Hungary
* Correspondence: panyi@med.unideb.hu; Tel.: +36-52-352201
† These authors contributed equally to this work.

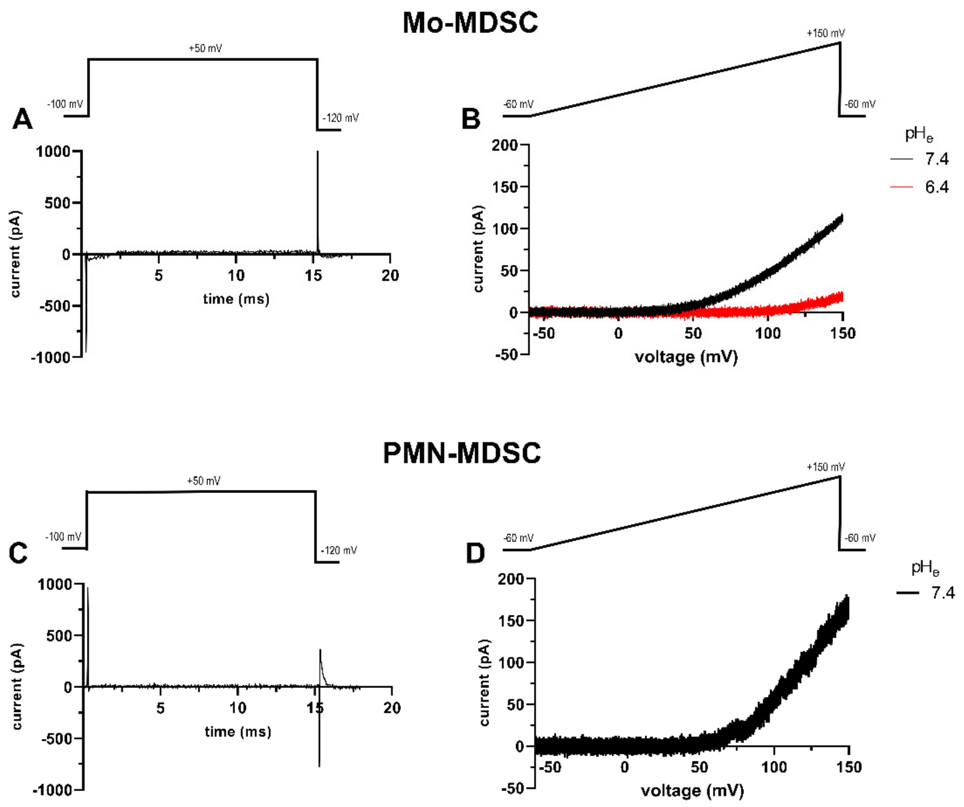

Supplementary Figure S1. Ion currents in physiological salt solutions. Whole-cell patch-clamp records were obtained in Na^+-based extracellular and K^+-based pipette filling solutions (see Materials and Methods for details). The pH of the pipette filling solution was pH_i=7.2 and that of the extracellular solution was pH_e=7.4 (black traces) or pH_e=6.4 (red trace). Solution exchange was performed as described in the Materials and Methods. Voltages steps (A and C) from the holding potential of -100 mV to +50 mV or voltage ramps (B and D) from the holding potential of -60 mV to +150 mV (ramp duration: 2s) were applied to elicit currents (see insets). Currents were recorded every 15 s. current traces are shown for Mo-MDSCs (A-B), and for PMN-MDSCs (C-D).

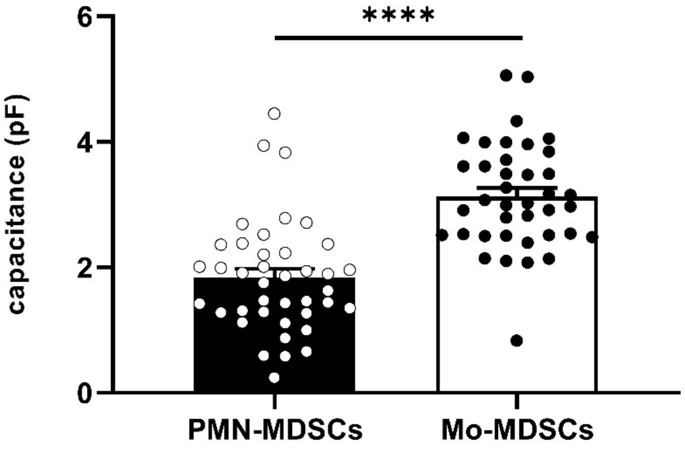

Supplementary Figure S2. Whole-cell capacitance of MDSCs. The cell membrane capacitance values were obtained from the readings of the whole-cell capacitance compensation circuit of the patch-clamp amplifier. The capacitances were determined after breaking into the whole-cell configuration. Each point represents an individual cell, bar heights represent mean, error bars are ± SEM. **** means $p < 0.0001$.

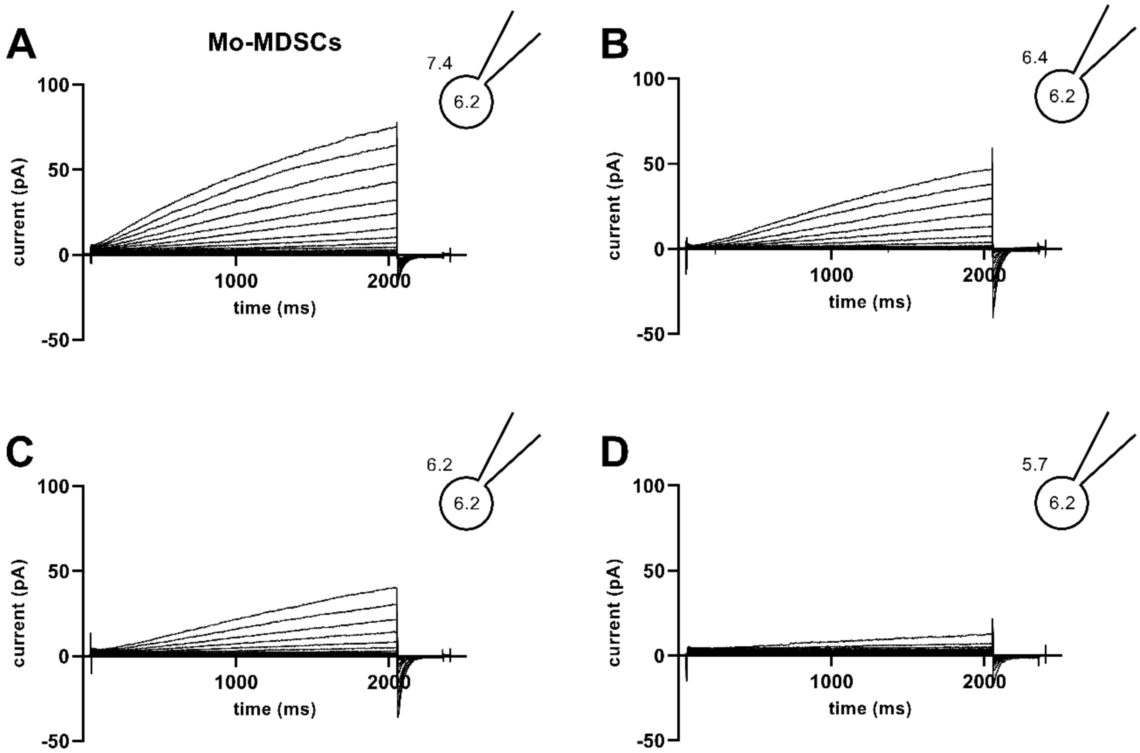

Supplementary Figure S3. Families of H^+ currents in a Mo-MDSC. Whole-cell H^+ currents were recorded in a murine Mo-MDSC freshly isolated from an LLC tumor. 2-s-long depolarizing pulses were applied from a holding potential of -80 mV to +100 mV in 10 mV increments. While the internal solution was maintained constantly in the micropipette at pH_i = 6.2, the cell was locally perfused at different pH_e values (7.4 (A), 6.4 (B), 6.2 (C) and 5.7 (D)). Representative of 5 (A), 5 (B), 4 (C) and 5 (D) similar families of currents. Every trace has been filtered with a 25-point boxcar filter.
